# Exploring Mediators of a Guided Web-Based Self-Help Intervention for People With HIV and Depressive Symptoms: Randomized Controlled Trial

**DOI:** 10.2196/12711

**Published:** 2019-08-23

**Authors:** Sanne van Luenen, Vivian Kraaij, Philip Spinhoven, Tom F Wilderjans, Nadia Garnefski

**Affiliations:** 1 Section of Clinical Psychology, Institute of Psychology Faculty of Social and Behavioural Sciences Leiden University Leiden Netherlands; 2 Department of Psychiatry Leiden University Medical Center Leiden Netherlands; 3 Section of Methodology and Statistics, Institute of Psychology Faculty of Social and Behavioural Sciences Leiden University Leiden Netherlands; 4 Leiden Institute for Brain and Cognition Leiden Netherlands; 5 Research Group of Quantitative Psychology and Individual Differences Faculty of Psychology and Educational Sciences KU Leuven Leuven Belgium

**Keywords:** HIV, depression, internet, cognitive behavioral therapy, coaching, randomized controlled trial, mediators

## Abstract

**Background:**

Cognitive behavioral therapy (CBT) is frequently used to treat depressive symptoms in people living with HIV. We developed an internet-based cognitive behavioral intervention for people with HIV and depressive symptoms, which was based on an effective self-help booklet. The Web-based intervention was previously found to be effective.

**Objective:**

The objective of this study was to investigate potential mediators of the Web-based intervention.

**Methods:**

This study was part of a randomized controlled trial, in which the intervention was compared with an attention-only waiting list control condition. Participants were 188 (97 in intervention group and 91 in control group) people with HIV and mild to moderate depressive symptoms recruited in HIV treatment centers in the Netherlands. A total of 22 participants (22/188, 11.7%) in the study were female and 166 (166/188, 88.3%) were male. The average age of the participants was 46.30 years (SD 10.63). The intervention comprised Web-based self-help CBT for 8 weeks, 1 to 2 hours a week, including minimal telephone support from a coach. The participants received Web-based questionnaires at pretest, 3 times during the intervention/or waiting period, and post intervention. The outcome was depressive symptoms. Factors tested as potential mediators were changes in behavioral activation, relaxation, the cognitive coping strategies catastrophizing and positive refocusing, goal re-engagement, and coping self-efficacy.

**Results:**

Using multilevel structural equation modeling, changes in behavioral activation (P=.006) and goal re-engagement (P=.009) were found to be significant mediators of the intervention effect. The mediation effect seemed to occur between weeks 3 and 5 for behavioral activation and weeks 1 and 3 for goal re-engagement. Using (bivariate) autoregressive latent trajectory analysis, we found a return effect (from the dependent variable to the mediator) for goal re-engagement but not for behavioral activation, which suggested that the mediation effect of changes in behavioral activation was stronger than that in goal re-engagement.

**Conclusions:**

The results suggest that changes in behavioral activation and goal re-engagement may mediate the effect of the Web-based intervention for people with HIV and depressive symptoms. The results may lead to possible mechanisms of change of the intervention and improvement of therapy outcomes.

**Clinical Trial:**

Netherlands Trial Register NTR5407; https://www.trialregister.nl/trial/5298

## Introduction

### Psychological Treatment for People Living With HIV and Depressive Symptoms

Depressive symptoms are quite common among people living with HIV (PLWH). This may be related to psychosocial factors, such as the stigma that is associated with having HIV [1], concerns about disclosing the illness to others [2], and difficulties with coping with HIV [3]. Symptoms of depression in PLWH may be treated with psychological interventions. Cognitive behavioral therapy (CBT) is frequently used to treat depressive symptoms in PLWH, and numerous studies have found it to be effective [4-8]. In CBT, the focus is on identifying and changing maladaptive cognitions and behaviors to improve one’s mood [9]. CBT provided through the internet is increasingly being used and investigated, and it was found to be equally successful as face-to-face CBT for people with depressive symptoms [10,11]. Internet-based treatments have some advantages over face-to-face treatments, such as their larger reach, lower costs, and increased accessibility. We developed an internet-based intervention with coaching for PLWH and depressive symptoms, which was based on an effective self-help booklet: *Living Positive With HIV* [12]. It has been found that this Web-based intervention was effective in treating depressive symptoms in PLWH, compared with a control group that received minimal coaching [13].

However, we do not know which factors are mediators of the intervention effect. Mediators are factors that (partially) explain the relation between an independent and a dependent variable. In this case, we look for treatment factors that may explain the relationship between receiving the Web-based intervention and the decrease in depressive symptoms [14]. When a mediator of intervention effect is found, it may provide us indications for possible mechanisms of change [15]. A mechanism of change is defined as a process that leads to change, which may answer the important question: how does the intervention work? It is important to have more knowledge about the mechanisms of change to be able to adapt and improve the intervention to optimize the outcome [15]. To investigate mediators of treatment outcome, at least 3 measurement moments are needed to establish a timeline of mediators and outcomes.

### Previous Research

Research on Web-based CBT to treat depressive symptoms in PLWH is scarce. As far as we know, no studies were conducted on mediators of Web-based CBT for depressive symptoms in PLWH, although potential mediators of CBT (face-to-face and Web-based) for people with depressive symptoms in general have been investigated in the last decade. First of all, when we look at face-to-face CBT for depressive symptoms, the literature regarding changes in cognitions as a mediator is mixed. A total of 3 reviews have found that a change in cognitions was an important mediator [16-18], whereas another review has concluded that there is little evidence for cognitive mediation in CBT for depression [19]. Therefore, the role of changing cognitions as a mediator in CBT for depressive symptoms is still unclear. Furthermore, the mediating role of behavioral factors such as changes in activation level in CBT for depression was investigated in a review [18]. Changes in behavioral factors were found to be a significant mediator in 3 out of 6 studies.

Next to mediation studies of face-to-face CBT for depressive symptoms, mediators were also investigated in Web-based CBT for depressive symptoms. It has been found that changes in dysfunctional attitudes, a negative problem orientation [20], repetitive negative thinking [20,21], use of cognitive skills [22], and perceived control over things in life [20,23] were mediators in the relation between Web-based CBT and (a decrease in) depressive symptoms. Increasing activity levels was not found to be a mediator in Web-based CBT for depression [22]. Concluding, the results regarding mediators of change of (Web-based) CBT are mixed and should be investigated further. In addition, many previous mediation studies correlated across subjects changes over time—using only 2 measurement moments (ie, pretest and posttest)—in 2 variables, which does not allow to establish a timeline of mediators and outcomes [15,18]. More research with at least 3 measurement moments is needed to establish this timeline.

### This Study

In this study, potential mediators of the effect of the Web-based intervention *Living Positive With HIV* on depressive symptoms were investigated. The intervention is based on CBT and contains 4 main components: behavioral activation, relaxation, changing negative thoughts into more balanced thoughts, and goal attainment. We statistically explored mediators for the decrease in depressive symptoms, which might refer to causal mechanisms of change that might have been activated by the intervention components. The following potential mediators were investigated in this study: changes in behavioral activation, relaxation, the cognitive coping strategies catastrophizing and positive refocusing, goal re-engagement, and coping self-efficacy. These mediators were investigated because they correspond with the components of the intervention. For example, learning to use adaptive cognitive coping strategies (potential mediator) was expected to be related to changes in cognitions (intervention component). We attempted to determine a temporal pattern of change: the mediators and the outcome (depressive symptoms) were investigated at pretest, 3 times during the intervention, and post intervention.

## Methods

### Participants and Procedure

This study is part of a randomized controlled trial (RCT; Netherlands Trial Register NTR5407) investigating the effectiveness of the self-help intervention *Living Positive With HIV*. More information about the procedure of the RCT can be found elsewhere [12]. Nursing consultants and doctors in 23 of 26 HIV treatment centers in the Netherlands recruited participants during regular checkups. Patients were screened with the Patient Health Questionnaire–2 (PHQ-2 [24]), and when their score was higher than 0, they were informed about the study and referred to the researchers when they were interested. Researchers called the patients and screened them on the inclusion criteria: being HIV positive for at least 6 months, aged >17 years, mastery of the Dutch or English language, available for the next 8 weeks, having internet and an email address, no current use of antidepressants or current use for >3 months without change of type or dose of antidepressants in the past 3 months, absence of severe cognitive impairments, not currently treated by a psychologist or psychiatrist, presence of mild to moderate depressive symptoms (determined by a PHQ-9 [25] score >4 and <20), and absence of severe suicide ideation (determined by a score <2 on question 9 of the PHQ-9).

When patients were eligible and agreed to participate, Web-based informed consent was signed. Thereafter, participants completed the pretest and were randomly allocated to the intervention or control condition (waiting list and attention-only from a coach). Stratified randomization by sex and HIV treatment center was performed. A random number table was used to create the sequence, which was done by an independent researcher and concealed from the main researcher. There were multiple measurement moments after randomization: 3 times during the intervention (lessons 1, 3, and 5) or waiting period (weeks 1, 3, and 5), a posttest when participants were finished with the intervention (experimental group) or 8 weeks after pretest (control group), and a follow-up at 3 and 6 months (the last follow-up was only completed in the intervention group). In this study, the follow-up measurements were not used in the analyses. Participants received €25 when they completed all questionnaires. The study was approved by the medical ethics committee of the Leiden University Medical Center (nr. P14.091).

### Study Conditions

#### Guided Web-Based Self-Help Intervention

The intervention comprises CBT and contains 4 main components. The first component is behavioral activation: participants are asked to think of a small positive activity that they can perform in the coming weeks (eg, taking a short walk, week 1). They are encouraged to engage in this activity and expand this to other activities. The second component is relaxation exercises that are available on the Web and take about 20 min (week 2). The third component is changing negative cognitions into more balanced cognitions (by challenging negative thoughts) and eliciting strong and positive feelings when negative feelings are experienced (counterconditioning, weeks 3-5). The last component is goal attainment: setting important, realistic, concrete personal goals (eg, quit smoking) and working on attaining them by increasing self-efficacy (weeks 6-7). The participants received log-in details for the secured website of the intervention. No changes to the intervention were made during the RCT. The intervention comprises 8 lessons with psychoeducation, exercises, and assignments. The participants were engaged with the intervention for approximately 8 weeks. On the basis of a pilot study, it was expected that they spent 1 to 2 hours a week on the intervention.

In addition, they were called by a personal coach each week for about 15 min. The coach checked the well-being of the participant and discussed the progress of the intervention. The coaches used motivational interviewing to motivate the participants to continue with the intervention to minimize attrition. Coaching was provided until the participant had finished the intervention, for a maximum of 10 weeks. When participants had not finished by then, they could complete the intervention on their own. The coaches were master’s degree students in clinical psychology or graduates with a master’s degree in the field of psychology. They received a training and followed a coaching manual. In the coaching manual, the questions that the coaches were supposed to ask were listed in the form of an example conversation to ensure that all coaches provided the same type of support. Furthermore, each coach was asked to record 2 calls in the beginning of the study, which were examined by the main researcher on adherence to the coaching manual. Weekly supervision sessions of 1 hour with coaches and a researcher to discuss issues encountered during coaching were scheduled in the beginning of the study. There were less issues at the end of the study; therefore, they were handled via email or phone. More information about the study conditions and procedures can be found elsewhere [12].

#### Control Condition

Participants that were allocated to the control condition were put on a waiting list and received attention only from a personal coach. Telephone coaching was provided for 8 weeks, approximately 5 min per week. The coach addressed the well-being of the participant, monitored depressive symptoms, and motivated the participant to keep waiting and complete questionnaires. The participant was referred to the HIV treatment center or general practitioner when the depressive symptoms worsened and became severe. After the 3-month follow-up, participants were invited to start with the intervention.

### Assessments

All assessments were completed on the Web and administered at pretest, weeks 1, 3, and 5 during the intervention and waiting period, and at posttest. The questions asked during the intervention and waiting period concerned the symptoms experienced during the last week. It is important to note that the measurements of weeks 3 and 5 and the posttest also capture the lessons learned in the weeks before, so it was not possible to solely measure pre to post session changes. To reduce the time to complete the questionnaires (it is approximately 10 min), 1 or 2 items were chosen from each questionnaire (with the chosen items being the same across measurement moments). The authors jointly determined the items that represented the concept the best. The decisions were made based on face validity. A confirmatory factor analysis (CFA) in R version 3.6.1 (the R foundation) was conducted to investigate whether the items of each questionnaire belonged to the same factor. A total of 7 factors were specified (1 for each mediator and the outcome measure). The CFA model was considered as fitting well when (1) the comparative fit index (CFI) and the Tucker–Lewis index (TLI) were >0.95, (2) the root mean square error of approximation (RMSEA) was <0.06, and (3) the 90% confidence interval for RMSEA had an upper bound <0.08 and a lower bound near 0 (not worse than 0.06) [26,27]. The fit indices show that the model was fitting well (CFI=0.98; TLI=0.96; RMSEA=0.05; 90% CI <0.001-0.07). The items load on the factors to which they belong and the correlations between most factors were low. The questionnaires are explained briefly below. More information on the specific questions used and the scoring can be found in Multimedia Appendix 1.

### Outcome Measure

The outcome measure for the mediational analysis was the severity of depressive symptoms. This was measured with the PHQ-2 [24] that comprises the first 2 questions of the PHQ-9. The construct and criterion validity of the PHQ-2 are adequate [24], and the Spearman–Brown coefficient ranged from 0.71 to 0.83 throughout the 5 measurement moments in this study. Item-total correlations of the 2 items were 0.42 and 0.55 at pretest.

### Potential Mediators

#### Activation

Behavioral activation was measured by a sum score of 2 items from the subscale activation of the Behavioral Activation for Depression Scale (BADS) [28]. The psychometric properties of the Dutch BADS are adequate [29], and the Spearman–Brown coefficient of the 2 items ranged from 0.79 to 0.84 throughout the 5 measurement moments in this study. Item-total correlations of the 2 items at pretest were 0.64 and 0.69.

#### Relaxation

Relaxation was measured with 1 self-designed item concerning difficulty to relax. The item-total correlation at pretest was 0.44. The reliability of this instrument could not be calculated because it comprised only 1 item.

#### Cognitive Coping: Catastrophizing and Positive Refocusing

The subscales catastrophizing and positive refocusing of the Cognitive Emotion Regulation Questionnaire short version (CERQ-short) [30] were adopted to measure the use of these cognitive coping strategies when thinking about having HIV. The subscales comprise 2 items each. The psychometric properties of the CERQ-short are adequate [30]. In this study, the Spearman–Brown coefficient ranged from 0.84 to 0.94 throughout the 5 measurement moments for the catastrophizing subscale and from 0.72 to 0.81 throughout the 5 measurement moments for the positive refocusing subscale. Item-total correlations were 0.72 and 0.82 at pretest for the catastrophizing subscale and 0.70 and 0.80 for the positive refocusing subscale.

#### Goal Re-Engagement

An item of the Goal Disengagement and Goal Re-engagement Scale (GDGRS) [31] was used to measure goal re-engagement. For this study, the item was specifically reformulated to measure goal re-engagement in relation to having HIV. The reliability of the total instrument was previously found to be satisfactory [31]. Item-total correlation at pretest was 0.80.

#### Coping Self-Efficacy

A sum score of 2 self-designed items was used to measure self-efficacy to cope with having HIV. The items were based on the Generalized Self-Efficacy Scale, which has good reliability and validity [32]. The Spearman–Brown coefficient of the 2 items in this study ranged from 0.75 to 0.92 throughout the 5 measurement moments. Item-total correlations of the 2 items were 0.73 and 0.79 at pretest.

### Statistical Analysis

The mediation analyses were conducted with the PHQ-2 score as dependent variable (Y), group (intervention and control) as independent variable (X), and activation, relaxation, the cognitive coping strategies catastrophizing and positive refocusing, goal re-engagement, and coping self-efficacy as potential mediators (M). Note that the PHQ-2 and all 6 mediator questionnaires were administered at all 5 measurement moments (pretest, weeks 1, 3, and 5, and posttest).

The mediation analyses were performed in 3 steps. In step 1, all potential mediators were entered separately into a multilevel structural equation model (MSEM [33]). An MSEM model was chosen because group (X) does not change over time (level 2: between-subjects level), whereas PHQ-2 (Y) and the mediator (M) scores do change over time (level 1: within-subjects level). As group (X) is constant over time, only mediation at between-subjects level can take place. To test this, MSEM computes the product term *a × b* and evaluates its significance, with *a* being the between effect from X to M and *b* the between effect from M to Y. Mediation is present when the product term significantly differs from 0. The significant mediators found were, thereafter, all together included in a single model to investigate which mediation effects remained significant after controlling for the other mediators in the model. The analysis in step 1 was repeated for the per protocol sample, as a sensitivity analysis. The per protocol sample included participants in the intervention group that finished at least the first 5 lessons of the intervention and participants in the control group that received at least 5 telephone calls from the coach.

In step 2, an explorative analysis was conducted to investigate when the mediating effect(s) exactly occurred (ie, in between which 2 measurement moments). To this end, for the significant mediators encountered in step 1, the same MSEM model was fitted as in step 1, however, using different combinations of measurement moments. In particular, the timing of the mediation effect(s) was investigated by comparing for each measurement moment an MSEM model including only the measurements up to that moment (including the measurement moment in question) with an MSEM model including only subsequent measurement moments. For example, for week 1, an MSEM model including the pretest and week 1 was compared with an MSEM model including weeks 3 and 5 and the posttest. The first measurement moment for which in both associated MSEM models mediation was present was considered as the moment when the mediation occurred.

In step 3, return effects from the dependent variable to the significant mediators identified in step 1 (ie, from Y to M) were studied. When return effects are present, this may indicate that the mediation effect is less strong. Return effects were investigated by means of a bivariate autoregressive latent trajectory analysis (ALT [34,35]). To get a good fitting but not too complex ALT model (which generalizes well), some constraints on the parameters were imposed. In particular, for each variable, parameters representing auto-regressive paths were set equal to each other, with the same being true for cross-lagged parameters. Furthermore, for each variable, residual variances for each measurement moment were kept equal (except for the first measurement moment as prescribed by the predetermined model parameterization [34]). Finally, time-specific correlations between residuals were set equal over time. To determine whether the ALT model fitted well to the data, the following model fit indices were evaluated: RMSEA with its 90% CI, CFI, and TLI. The model has a good fit when the RMSEA value is below 0.06, when the 90% CI for RMSEA has an upper bound <0.08 and a lower bound close to 0 and when the CFI and TLI values are higher than 0.95 [26,27]. The analyses were based on full information maximum likelihood techniques, which means that all available data, including participants with partially missing data, were used. Alpha=.05 was used for significance testing. All analyses were conducted in MPlus version 7.31.

## Results

### Participants

**Figure 1:**
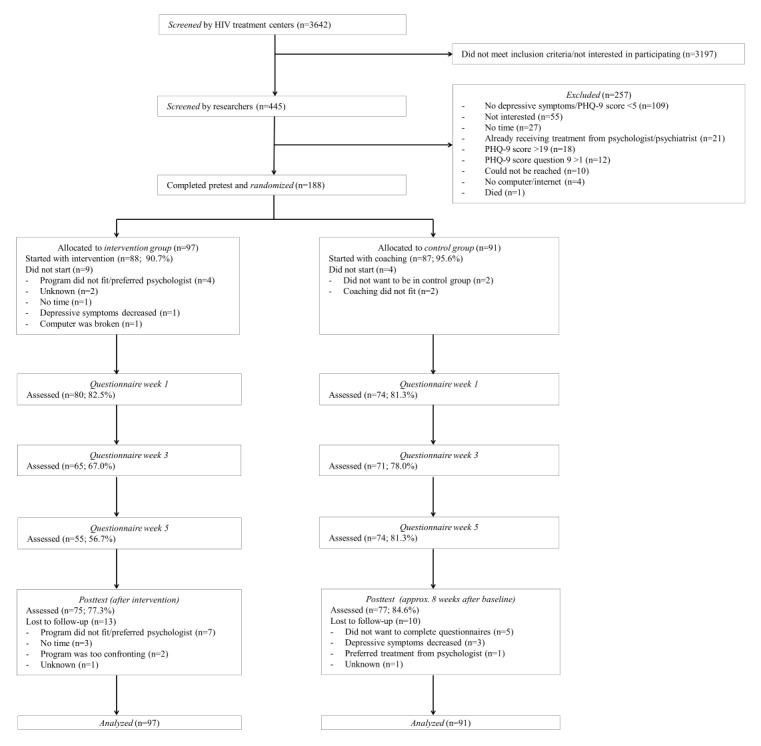
Flow of participants through the study. PHQ-9: Patient Health Questionnaire-9.

In the HIV treatment centers, 3642 patients were screened on depressive symptoms. Of these, 445 were screened by the researchers and 188 patients were included in the study. Patients were 1:1 randomized to the intervention group (n=97) and the control group (n=91). Note that because of the stratified randomization, the intervention group contains a few more participants than the control group. The posttest was completed by 75 participants (75/97, 77%) of the intervention group and 77 participants (77/91, 84%) of the control group. Figure 1 displays for each group separately the flow of participants through the study in terms of PHQ-2.

In total, 22 participants (22/188, 11.7%) in the study were female and 166 (166/188, 88.3%) were male. In the Netherlands, most PLWH are male. The average age of the participants was 46.30 years (SD 10.63). Most participants had a Dutch nationality (158/188, 84.0%), 18 participants (18/188, 9.6%) had another nationality (most common Surinamese), and 12 (12/188, 6.4%) had a Dutch nationality combined with another nationality. A total of 32 participants (32/188, 17.0%) were heterosexual, 144 (144/188, 76.6%) homosexual, and 12 (12/188, 6.4%) bisexual. Most participants had a medium education (77/188; 40.9%) or a high education (69/188, 36.7%), and a minority had a low education (42/188, 22.3%; classification of educational level according to Statistics Netherlands [36]). Participants had HIV for 9.87 years on average (SD 6.58). A total of 23 participants (23/188, 12.2%) had AIDS and 165 (165/188, 87.8%) did not have AIDS, and 184 participants (184/188, 97.9%) used antiretroviral therapy (ART) and 4 (4/188, 2.1%) did not use ART. More information on the baseline characteristics of the sample can be found elsewhere [13]. Mean scores on the questionnaires at different time points can be found in Table 1.

**Table 1:** Mean scores on the questionnaires at different time points.

Characteristic		Intervention group, mean (SD)	Control group, mean (SD)	Mean difference	*P* value
**Depressive symptoms (PHQ-2^**a**^) pretest**		3.10 (1.47)	2.78 (1.26)	0.32	.11
	Week 1	2.13 (1.41)	2.27 (1.55)	−0.15	.54
	Week 3	1.66 (1.20)	2.24 (1.56)	−0.58	.02
	Week 5	1.24 (1.25)	2.05 (1.59)	−0.82	.002
	Posttest	1.53 (1.41)	2.38 (1.59)	−0.84	.001
**Behavioral activation (BADS^b^) pretest**		4.78 (3.15)	4.58 (3.05)	0.20	.66
	Week 1	5.90 (2.74)	5.30 (2.88)	0.60	.19
	Week 3	6.52 (2.88)	5.35 (2.82)	1.17	.02
	Week 5	7.35 (2.31)	5.78 (2.74)	1.56	.001
	Posttest	7.57 (2.99)	5.46 (3.07)	2.11	<.001
**Relaxation pretest**		1.62 (0.60)	1.69 (0.68)	−0.07	.43
	Week 1	1.66 (0.57)	1.74 (0.62)	−0.08	.40
	Week 3	1.97 (0.59)	1.80 (0.65)	0.17	.12
	Week 5	2.13 (0.70)	1.81 (0.61)	0.32	.01
	Posttest	2.05 (0.66)	1.86 (0.70)	0.20	.08
**Catastrophizing (CERQ-short^c^) pretest**		3.80 (2.36)	3.37 (1.74)	0.43	.15
	Week 1	3.44 (1.77)	3.61 (2.10)	−0.17	.60
	Week 3	3.05 (1.58)	3.38 (2.04)	−0.33	.29
	Week 5	2.75 (1.21)	3.24 (1.68)	−0.50	.05
	Posttest	2.92 (1.75)	3.16 (1.69)	−0.24	.39
**Positive refocusing (CERQ-short) pretest**		6.39 (2.23)	6.37 (2.03)	0.02	.95
	Week 1	6.13 (1.89)	5.96 (1.85)	0.17	.58
	Week 3	6.32 (1.96)	5.90 (1.97)	0.42	.21
	Week 5	6.91 (1.96)	6.39 (2.05)	0.52	.15
	Posttest	7.04 (2.17)	6.07 (2.19)	0.97	.01
**Goal re-engagement (GDGRS^d^) pretest**		3.15 (0.86)	2.97 (0.82)	0.19	.13
	Week 1	3.42 (0.79)	3.12 (0.81)	0.30	.02
	Week 3	3.55 (0.75)	3.10 (0.94)	0.46	.002
	Week 5	3.71 (0.79)	3.26 (0.97)	0.45	.01
	Posttest	3.55 (0.79)	3.08 (0.99)	0.47	.002
**Coping self-efficacy pretest**		7.08 (1.78)	7.24 (1.66)	−0.16	.53
	Week 1	7.51 (1.59)	7.15 (1.78)	0.36	.19
	Week 3	8.03 (1.60)	7.14 (1.83)	0.89	.003
	Week 5	8.11 (1.40)	7.53 (1.69)	0.58	.03
	Posttest	7.93 (1.65)	7.20 (1.72)	0.73	.02

^a^PHQ-2: Patient Health Questionnaire–2.

^b^BADS: Behavioral Activation for Depression Scale.

^c^CERQ-short: Cognitive Emotion Regulation Questionnaire short version.

^d^GDGRS: Goal Disengagement and Goal Re-engagement Scale.

### Mediation Analysis Step 1

**Figure 2:**
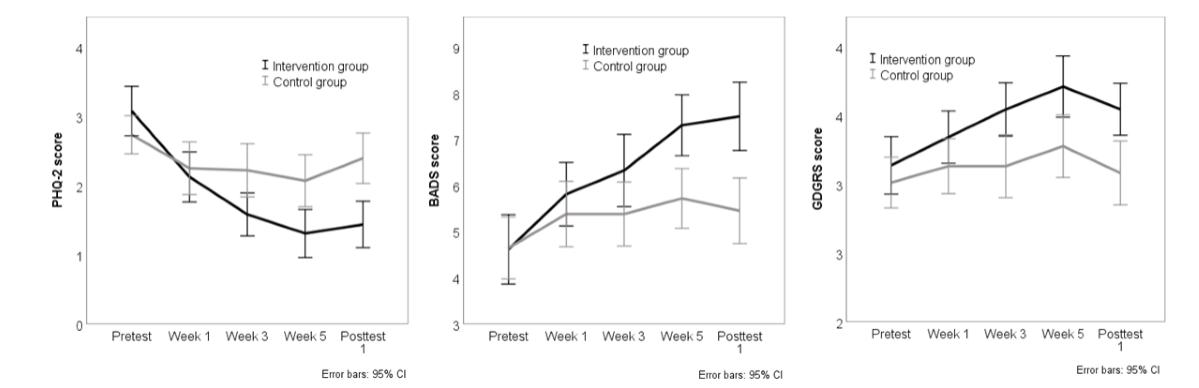
Course of the mean Patient Health Questionnaire–2 (PHQ-2) score, Behavioral Activation for Depression Scale (BADS) score, and Goal Disengagement and Goal Re-engagement Scale (GDGRS) score over time for both groups (per protocol sample).

Table 2 shows the results of the mediation analysis based on MSEM in which all mediators are investigated separately. Changes in BADS and GDGRS were found to be significant mediators. Subsequently, these 2 mediators were together included in a single model. Changes in BADS remained a significant mediator when changes in GDGRS were controlled for (*a × b*=0.25; SE 0.10; *P*=.01), whereas changes in GDGRS were not a significant mediator anymore when changes in BADS were controlled for (*a × b*=0.15; SE 0.09; *P*=.10). Correlations between BADS and GDGRS varied across measurement moments (range from *r*=0.07; *P*=.39 to *r*=0.54; *P*<.001). The mediation analysis was repeated on the per protocol sample and the results were similar as for the whole sample. For illustrative purposes, Figure 2 displays the course of PHQ-2, BADS, and GDGRS scores over time in both groups. The intervention group shows a stronger reduction in PHQ-2 score over time than the control group, and at the same time BADS scores and GDGRS scores increase more over time in the intervention group than in the control group.

**Table 2:** Mediation effects of 6 potential mediators (tested separately) with group as independent variable and Patient Health Questionnaire-2 score as dependent variable, based on multilevel structural equation model analysis on data containing all 5 measurement moments.

Potential mediator	*a × b^a^*	SE	*P* value
Behavioral activation (BADS^b^)	0.31	0.11	.006^c^
Relaxation	0.05	0.08	.55
Catastrophizing (CERQ-short^d^)	−0.005	0.06	.92
Positive refocusing (CERQ-short)	0.10	0.08	.17
Goal re-engagement (GDGRS^e^)	0.29	0.11	.009^c^
Coping self-efficacy	0.12	0.08	.13

^a^Coefficient for the product term testing the mediation effect.

^b^BADS: Behavioral Activation for Depression Scale.

^c^*P*<.05.

^d^CERQ-short: Cognitive Emotion Regulation Questionnaire short version.

^e^GDGRS: Goal Disengagement and Goal Re-engagement Scale.

### Mediation Analysis Step 2: Timing of Mediation Effects

Table 3 shows the timing of the mediation effects of the significant mediators in step 1. The results show that for changes in BADS, the mediation effect is not significant when the pretest and weeks 1 and 3 measurements are combined. However, the mediation effect is significant when the week 5 and posttest measurements are combined. Therefore, it seems likely that the BADS mediation effect occurs between weeks 3 and 5. For changes in GDGRS, the results are almost similar. The mediation effect is not significant when the pretest and week 1 measurements are combined and is significant when the weeks 3 and 5 and posttest measurements are combined. Hence, it seems likely that the GDGRS mediation effect occurs between weeks 1 and 3.

**Table 3:** Timing of mediation effects—comparison of the mediation effect in multilevel structural equation models fitted to data containing different sets of measurement moments to investigate when the mediation effect occurs.

Combination of measurement moments		BADS^a^			GDGRS^b^		
		*a × b^c^*	SE	*P* value	*a × b*	SE	*P* value
**Combination 1**							
	Pretest	0.03	0.06	.66	0.06	0.04	.17
	Week 1–posttest	0.44	0.13	.001^d^	0.43	0.14	.003^d^
**Combination 2**							
	Pretest–week 1	0.10	0.10	.30	0.20	0.12	.09
	Week 3–posttest	0.59	0.16	<.001^d^	0.50	0.18	.005^d^
**Combination 3**							
	Pretest–week 3	0.20	0.11	.08	0.19	0.10	.04^d^
	Week 5–posttest	0.69	0.20	<.001^d^	0.60	0.27	.03^d^
**Combination 4**							
	Pretest–week 5	0.23	0.11	.04^d^	0.22	0.10	.02^d^
	Posttest	0.52	0.15	<.001^d^	0.24	0.10	.02^d^

^a^BADS: Behavioral Activation for Depression Scale.

^b^GDGRS: Goal Disengagement and Goal Re-engagement Scale.

^c^Coefficient for the product term testing the mediation effect.

^d^*P*<.05.

### Mediation Analysis Step 3: Return Effects

Table 4 presents the results of the analysis on return effects from the dependent variable to the significant mediators. The values of the fit indices (RMSEA, CFI, and TLI) indicate that the model has an acceptable to good fit. The results show that there is no return effect from the PHQ-2 to the BADS, but there is a return effect from the PHQ-2 to the GDGRS. However, the standardized coefficient (beta) for the effect of the GDGRS on the PHQ-2 (beta=−.20) is higher, in absolute value, than the beta for the effect of the PHQ-2 to the GDGRS (beta=−.13). This suggests that the mediation effect is larger than the return effect.

**Table 4:** Results of the analysis on return effects from the dependent variable (Patient Health Questionnaire–2) to the mediators.

Mediator	Dependent variable → Mediator		*P* value	RMSEA^a^ (90% CI)	CFI^b^	TLI^c^
	Unstandardized coefficient (SE)	Standardized^d^ coefficient (SE)				
BADS^e^	−0.12 (0.10)	−0.06 (0.05)	.24	0.06 (0.04-0.09)	0.94	0.94
GDGRS^f^	−0.08 (0.03)	−0.13 (0.06)	.02^g^	0.06 (0.03-0.08)	0.95	0.94

^a^RMSEA: root mean square error of approximation*.*

^b^CFI: comparative fit index.

^c^TLI: Tucker–Lewis Index.Using STDYX standardization.

^d^Using STDYX standardization.

^e^BADS: Behavioral Activation for Depression Scale.

^f^GDGRS: Goal Disengagement and Goal Re-engagement Scale.

^g^*P*<.05.

## Discussion

### Principal Findings

This study investigated potential mediators of a guided internet-based intervention for PLWH with depressive symptoms, compared with a control group that received attention only. Changes in behavioral activation and goal re-engagement were found to be significant mediators of the intervention effect. For changes in behavioral activation, the mediation effect seemed to occur between weeks 3 and 5 of the intervention and for changes in goal re-engagement, between weeks 1 and 3. The mediation effect of changes in behavioral activation seemed to be stronger than the effect of changes in goal re-engagement because goal re-engagement was not a significant mediator anymore when the model was controlled for behavioral activation. Moreover, a return effect (from the dependent variable to the mediator) was found for goal re-engagement and not for behavioral activation.

In a review about CBT for depression, changes in behavioral activation were found to be a significant mediator in 3 out of 6 studies [18]. More specifically, when only high-quality studies were examined, 3 out of 4 studies concluded that changes in behavioral factors were a significant mediator. This is in line with our findings. However, a previous study into internet CBT for depression investigated changes in behavioral activation as a mediator and found that it was not a significant mediator of the intervention effect [22]. An explanation for the difference in results between our study and this previous study may be the difference in timing of the intervention components and measurement moments, that is, the component behavioral activation was offered early in our intervention and late in the other intervention. We included 3 measurement moments during the intervention period in our study and there was only 1 measurement moment during the intervention in the previous study, and at that moment behavioral activation was not offered yet. Therefore, it is not surprising that no mediation effect of behavioral activation was found in the previous study. It is important to include multiple measurement moments of the dependent variable and possible mediators during the intervention period to determine a timeline of the effects of mediators and outcome. As far as we know, changes in goal re-engagement as a mediator of intervention effect for (Web-based) CBT for depression was not investigated previously. More research is needed regarding the mediating role of changes in behavioral activation and goal re-engagement in Web-based CBT for depressive symptoms.

This study was conducted to find mediators of the intervention effect, which may provide us with suggestions for possible mechanisms of change underlying the intervention. As changes in behavioral activation and goal re-engagement were found to be mediators of the intervention, they might suggest possible mechanisms of change. It was previously found that reward processing and avoidance might be possible mechanisms of change of behavioral activation [37-39], that is, specific components of the intervention might activate these mechanisms, for example, by activating participants, avoidance of (positive) activities might be reduced. In turn, this may lead to a reduction in depressive symptoms. However, it was previously found that there is no 1-to-1 relation between offering certain components of the intervention and the change in corresponding mediators [19,40]. For example, it was found that negative thinking decreased after CBT but also after behavioral activation. So, even when the focus was not on changing negative thoughts in the behavioral activation treatment, they did decrease [40]. Though, the results of this study may suggest that behavioral activation and goal re-engagement may be important components of the intervention. More research should be conducted into the relation between offering certain components of the intervention and the change in corresponding mediators.

Goal re-engagement and behavioral activation as components of the intervention are related, as both are trying to increase the amount of (positive) activities to improve one’s mood. In addition, in interventions that include behavioral activation, goal setting is often included as a first step of activation [41,42]. As behavioral activation and goal re-engagement are related, it may not be surprising that the timing of the mediation effects did not correspond to the timing of the related intervention components. The mediation effect of changes in behavioral activation occurred approximately 3 weeks after the component was introduced, and in goal re-engagement occurred approximately 4 weeks before the component was introduced. This is also in line with previous findings regarding the weak relation between offering a certain intervention component and a change in the corresponding mediator [19,40]. No other significant mediators of intervention effect were found. This means that changes in relaxation, coping self-efficacy, and the cognitive coping strategies catastrophizing and positive refocusing were no significant mediators. In most previous reviews [16-18], changes in cognitions were found to be mediators of CBT for depression, but 1 review found no evidence for changes in cognitions as a mediator [19]. In this study, changes in cognitions were not measured, but changes in the use of cognitive coping strategies was included. This may be comparable with a change in cognitions, but changes in the use of cognitive coping strategies were not found to be mediators. These cognitive coping strategies were addressed in the intervention and also did improve in the intervention group. However, the use of these strategies also improved in the control group. Future studies may investigate changes in cognitions as a mediator of intervention effect. Many PLWH suffer from depressive symptoms which are related to, among others, the stigma that is associated with having HIV [1] and coping difficulties [3]. The intervention that was investigated in this study was able to decrease depressive symptoms in PLWH. This may also have a positive effect on their quality of life and medication adherence. The findings from this study and future research may be used to optimize the intervention and improve the mental health of PLWH even more.

### Strengths and Limitations

Some strengths and weaknesses of this study may be identified. An important strength was that a temporal pattern of change was investigated because multiple measurement moments were included during the intervention period. Many previous mediation studies only included a pretest and a posttest, which is not sufficient to demonstrate a timeline and *real* mediation effects [15]. In addition, multiple mediators were investigated that corresponded to components of the intervention. Another strength was that advanced state-of-the-art statistical analyses were used: MSEM and ALT. Furthermore, all available data were used in the analyses, so participants with some missing measurement moments were not totally excluded from the analyses. Finally, return effects from the dependent variable to the mediators were investigated to study the strength of the mediation effects.

A weakness of this study is that the measurement of some mediators included the use of self-designed questionnaires with only a few items. The reliability and construct validity (established with factor analysis) of these questionnaires was mostly adequate, but the validity of the short scales needed to be more thoroughly investigated. Only a few items were used because multiple concepts were measured multiple times and it should not have taken too much time to complete them. Another weakness was that there was much dropout during the study. However, the dropout rate was comparable with other studies regarding the effectiveness of internet interventions [43,44]. No differences in demographic and HIV specific characteristics (eg, duration of HIV) were found between dropouts and completers in this study [13], so probably attrition bias was not a problem. Furthermore, the measurements of weeks 3 and 5 and the posttest also capture the lessons learned in the weeks before, so it was not possible to solely measure pre to post session changes. Finally, a selection of mediators was investigated in this study. Other mediators may also have an effect and may be assessed in future studies.

### Future Research

In future studies, mediators may be more elaborately assessed with validated questionnaires with more items. Attrition may be prevented by using techniques that were previously suggested, such as inducing hope for benefits of the intervention and reducing time barriers by using habit-forming strategies [45]. Other potential mediators may be investigated, such as changes in worrying. In addition, it is important to study what the mechanisms of change of the intervention are. This is a challenge to investigate, as the relation between intervention components, mediators, and mechanisms of change is weak. As a first step, dismantling studies may be conducted, where each component of the intervention is provided to a different group of participants and will be compared with a group that receives the complete intervention [15]. In this way, it can be investigated which components may be related to changes in specific mediators. Furthermore, manipulation of a proposed mechanism of change may be conducted to study the effects on the outcome [15]. Finally, it may be useful to conduct studies in minority groups in the Netherlands, such as females and heterosexual males with HIV, to investigate their specific needs. In addition, the gender distribution may be different in other countries, which has to be taken into account in future studies.

### Conclusions

To conclude, potential mediators of a guided internet-based intervention for PLWH with depressive symptoms were studied. The intervention was previously found to be effective in decreasing depressive symptoms, compared with a control group receiving attention only. We found that changes in behavioral activation and goal re-engagement were significant mediators of the intervention effect. The mediation effect of changes in behavioral activation seemed to be stronger than that in goal re-engagement. The mediators that were found in this study may suggest possible mechanisms of change of the intervention. More research into these mechanisms of change is needed to find out how the intervention works. The outcomes of these studies may be used to optimize the intervention and help decrease depressive symptoms among PLWH even more effectively.

## Appendix

Multimedia Appendix 1 Items and scoring of the questionnaires.

Multimedia Appendix 2 CONSORT-EHEALTH checklist (V 1.6.1).

## Abbreviations

ART: antiretroviral therapy
BADS: Behavioral Activation for Depression Scale
CBT: cognitive behavioral therapy
CERQ-short: Cognitive Emotion Regulation Questionnaire short version
CFA: confirmatory factor analysis
CFI: comparative fit index
GDGRS: Goal Disengagement and Goal Re-engagement Scale
MSEM: multilevel structural equation model
PHQ: Patient Health Questionnaire
PLWH: people living with HIV
RMSEA: root mean square error of approximation
TLI: Tucker–Lewis index
