# Supplementary material for: Exploring Mediators of a Guided Web-Based Self-Help Intervention for People With HIV and Depressive Symptoms: Randomized Controlled Trial
Source: JMIR Ment Health. 2019 Aug 23;6(8):e12711. doi: 10.2196/12711 (PMC6731054; doi:10.2196/12711)
Supplement: Multimedia Appendix 1 [file mental_v6i8e12711_app1.pdf]

Multimedia Appendix 1: Items and scoring of the questionnaires

Depressive symptoms (PHQ-2)

Over the last week, how often have you been bothered by any the following problems? Score from 0 (not at all) to 3 (nearly every day).

1 Little interest or pleasure in doing things.

2 Feeling down, depressed, or hopeless.

Activation (BADS)

Please indicate to what extent the following statements apply to you over the past week. Score from 0 (not at all) to 6 (completely).

1 I engaged in a wide and diverse array of activities.

2 I am content with the amount and types of things I did.

Relaxation

Please indicate which answer is most appropriate to you over the past week. Score 1 (yes), 2 (sometimes), 3 (no).

1 Is it difficult for you to relax?

Cognitive coping: catastrophizing and positive refocusing (CERQ-short)

Please read the sentences below and indicate how often you had the following thoughts over the past week. Score from 1 ((almost) never) to 5 ((almost) always).

1 I keep thinking about how terrible it is that I have HIV.

2 I continually think how horrible it is to have HIV.

3 I think of pleasant things that have nothing to do with having HIV.

4 I think of something nice instead of having HIV.

Goal reengagement (GDGRS)

We will ask you to click the answer to the statement that you think best suits you during the past week. Score from 1 (totally disagree) to 5 (totally agree).

1 If I have to stop pursuing an important goal in my life because I have HIV I start working on other new goals.

Coping self-efficacy

Click the answer that is most applicable to you over the past week. Score from 1 (totally disagree) to 5 (totally agree).

1 I am confident that I can deal with having HIV.

2 Whatever happens in relation to having HIV, I’ll figure it out.
